# lncRNA NEAT1 promotes the proliferation and metastasis of hepatocellular carcinoma by regulating the FOXP3/PKM2 axis

**DOI:** 10.3389/fonc.2022.928022

**Published:** 2022-09-15

**Authors:** Junping Pan, Yingzhe Hu, Chenlu Yuan, Yafu Wu, Xinhua Zhu

**Affiliations:** ^1^ Department of Hepatobiliary Surgery, Affiliated Drum Tower Hospital, Medical School of Nanjing University, Nanjing, China; ^2^ Department of Hepatobiliary Surgery, Nanjing Drum Tower Hospital Clinical College of Traditional Chinese and Western Medicine, Nanjing University of Chinese Medicine, Nanjing, China

**Keywords:** hepatocellular carcinoma, NEAT1, FOXP3, PKM2, long non-coding RNA

## Abstract

**Objective:**

Hepatocellular carcinoma (HCC) is a malignant tumor. The occurrence of HCC is involved in the alteration of a variety of oncogenes or tumor suppressor genes, but the specific molecular mechanism remains unknown. This research proved the effects of long non-coding RNA NEAT1 (lncRNA NEAT1) on the viability, proliferation, migration, and invasion of hepatocellular carcinoma cells and explored the mechanism behind these effects.

**Methods:**

NEAT1 in 97H and Huh7 cell lines was overexpressed or knocked down, respectively. The expression of FOXP3 and its target gene PKM2 was hinged on qRT-PCR and Western blot, respectively. RNA pulldown and RNA immunoprecipitation experiments were carried out to detect the interaction between NEAT1 and proteins. Finally, the effect of NEAT1 on the tumor volume of HCC was verified by animal experiments.

**Results:**

A series of experiments have shown that NEAT1 knockdown can inhibit the viability, proliferation, migration, and invasion of HCC cells; NEAT1 can bind FOXP3 to promote PKM2 transcription; PKM2 knockdown can inhibit the viability, proliferation, migration, and invasion of HCC cells; and PKM2 knockdown reversed the function of NEAT1.

**Conclusion:**

lncRNA NEAT1 can promote the malignant behavior of HCC cells, while silencing of NEAT1 can inhibit that behavior of HCC cells. Mechanically, NEAT1 promotes the transcriptional activation of PKM2 by binding FOXP3, and PKM2 knockout reverses the function of NEAT1.

## Introduction

Hepatocellular carcinoma (HCC) is the common reason of cancer death (1–3). At present, the treatment of HCC has made great progress, but the treatment in preventing metastasis and recurrence is still very limited (4). The pathogenesis of HCC is very complex, involving multiple genes and signaling pathways. Exploring the mechanisms of HCC progression is necessary to identify new therapeutic approaches (5).

Long non-coding RNA (lncRNA) is a structure with a 5'-cap and 3'-polyadenylated tail (6). lncRNA could bind with transcription factors, activators, inhibitors, or other target genes to regulate transcription and expression. lncRNAs are involved in a variety of biological processes (7, 8). lncRNA NEAT1 belongs to the lncRNA (9, 10). At present, lncRNA NEAT1 expression changes have been found in a variety of human tumors, and it is related to tumor metastasis (11), occurrence, and development (12). However, studies on lncRNA NEAT1 in HCC have been limited.

Earlier studies showed that FOXP3 was expressed in T cells (13). Later, it was also found to be expressed in kidney, breast, melanoma, and other cancer cells and tissues (14). FOXP3 regulates the expression of downstream genes by binding with the promoter region or other transcription factors and is involved in the biological functions of tumor cells (15). The study of FOXP3-related functions may display a target for the targeted therapy of HCC.

Pyruvate kinase has four isoenzymes, namely, PKM1, PKM2, PKL, and PKR. During tumor formation, other isoenzymes disappear and PKM2 is highly expressed, laying a new biological foundation for cancer treatment (16, 17). PKM2 is a pivotal protein associated with microenvironmental sugar metabolism, cell growth signals, and oxidative stress in cancer cells. In terms of aerobic glycolysis of cancer cells, PKM2 acts as a biofunctional sensor and regulator of glycolysis in the form of high-activity tetramer and low-activity dimer to determine whether it is metabolized into lactic acid or synthesized into biological macromolecules.

To find the lncRNA NEAT1 mechanism *in vitro* and *in vivo*, studies have shown that NEAT1, by binding FOXP3, regulates the transcriptional activation of PKM2. This study extends our understanding of the role lncRNAs play in HCC development and may provide novel targets for the clinical therapy of HCC.

## Materials and methods

### Materials

The primary antibodies used in the experiment were purchased from BD, USA, and the secondary antibodies were purchased from Invitrogen, USA. PcDNA3.1 carriers are provided by Shanghai Gemal Biotechnology Co., Ltd.,Shanghai, China. Lipofectamine™ 2000 transfection reagent was purchased from BD, USA. The RT-PCR instrument was purchased from BD, USA, and the HBS-1096B microplate was purchased from Nanjing Detie Experimental Equipment Co., Ltd. The Western blot electrophoresis equipment was purchased from Bio-Rad, USA.

### Experimental subject

In this study, HCC tissues were obtained from patients undergoing surgery at the Affiliated Drum Tower Hospital (*n* = 56), all of whom had received preoperative radiotherapy. These tissues were quick-frozen in liquid nitrogen and stored at −80°C until needed. Informed consent was been obtained from all patients. The scheme was approved by our research ethics committee.

### Cell culture, transfection, and grouping

Hepatocellular carcinoma cell lines including 97H and Huh7 were from the medical school and cultured in RPMI 1640 medium at 37°C in a 5% CO_2_ incubator. The siRNAs targeting NEAT1, FOXP3, and PKM2 as well as the negative control were purchased from GenePharma. The overexpressing vector of NEAT1 and PKM2 and the vector control were from GenePharma. The knockdown vector of Psilencer/PKM2 was synthesized by Hanbio. The plasmids were transfected into HCC cells using Lipofectamine 3000. The transfection concentration was 300 nmol/well. The cells were divided into the following groups: pcDNA3.1 (empty carrier), pcDNA3.1/NEAT1 (transfected NEAT1 plasmid), Si-nc (transfected with NEAT1 negative plasmid), Si-NEAT1 1# (transfected NEAT1 low-expression plasmid 1#), Si-NEAT1 2# (transfected NEAT1 low-expression plasmid 2#), vector (empty carrier), NEAT1 (transfected NEAT1 overexpression plasmid), Si-NEAT1 (transfected with NEAT1 low-expression plasmid), Si-FOXP3 1# (transfected FOXP3 low-expression plasmid 1#), Si-FOXP3 2# (transfected FOXP3 low-expression plasmid 2#), NEAT1 + Si-nc (NEAT1 group continued to transfect FOXP3-negative plasmid), NEAT1 + Si-FOXP3 (NEAT1 group continued to transfect FOXP3 low-expression plasmid), Ad-vector (adenovirus transfected with empty vectors), Ad-NEAT1 (adenovirus transfected with NEAT1), pcDNA3.1/PKM2 (transfected PKM2 plasmid), Si-PKM2 (transfected with PKM2 low-expression plasmid), and NEAT1 + Si-PKM2 (NEAT1 group continued to transfect PKM2 low-expression plasmid).

### MTT

HCC cells were inoculated into 96-well plates at a density of 1 × 10^4^ cells/well and incubated overnight at 37°C in a 5% CO_2_ incubator. Cells were treated with 0, 4, 20, 100, 500, and 2,500 μg/ml of the chemotherapeutic drug TMZ (Kaigi Company, Nanjing, China) in a medium containing 10% FBS for 24 h. Meanwhile, the IC_50_ of TMZ was detected. Then, 10 μl of MTT (0.5 mg/ml) was added to each well and incubated for 4 h. After removing the supernatant, 200 μl of dimethyl sulfoxide (DMSO) was added to stop the reaction and incubated at 37°C for 15 min. The absorbance at 490 nm was measured with a Bio-Rad microplate meter (Bio-Rad, Hercules, CA, USA).

### The cell colony formation experiment

After different treatments, the cells were digested and cultured in six-well plates for 2 weeks. Afterward, the cells were fixed with 10% formaldehyde and stained with 1% crystal violet. After washing, the stained cell colonies were observed and imaged (Leica, Germany). The number of cell clones was counted.

### Luciferase report

The PKM2 promoter was amplified from the cDNA of the HEK293T cell and subcloned into the luciferase reporter vector. HEK293T cells were co-transfected with 100 ng of luciferase vectors and other plasmids (50 nM). Cells were collected for 48 h.

### Biotinylated pulldown assay

The biotinylated pulldown assay was based on previous instructions. In brief, the biotin-coupled RNA complexes were captured and transfected with 50 μM of the biotinylated NEAT1 probe or the control probe. Streptavidin beads (100 µl) were added to each reaction tube, and the biotin-coupled RNA complexes were assembled at room temperature. Western blot was used to evaluate the expression of proteins bound to NEAT1.

### Transwell assay

For the migration assay, transfected 3 × 10^4^ HCC cells were inoculated on the upper surface of the Transwell compartment. RPMI 1640 medium containing 12% FBS was added to the lower cell chamber. The cells were incubated in an incubator at 37°C for 24 h. The cells under the septum were then fixed and stained with 0.2% crystal violet solution for 10 min. Ten random fields of view (×200) were examined and the number of cells under the septum was recorded. Regarding cell invasion, a Transwell chamber with a precoated 100-μl Matrigel was used.

### RNA extraction and qRT-PCR

RNA was extracted and separated using the All-in-One miRNA extraction kit and the All-in-One miRNA qRT-PCR assay kit following the instructions of the manufacturer. After RNA was extracted, the RNA concentration was determined by a NanoDrop 1000 spectrophotometer. Real-time PCR reaction was performed using an SYBR Green Mix kit and a FAST7500 Real-time PCR system. The gene sequences are shown in **Table 1**. The 2^−ΔΔCt^ quantitative method was used to calculate the relative expression of the genes.

**Table 1:** qRT-PCR primer.

Gene	Primer sequence (5'–3')
***NEAT1***	F: GTTCCGTGCTTCCTCTTCTG R: GTGTCCTCCGACTTTACCAG
***FOXP3***	F: CTCCAATCCCTGCCCTTGACC R: ACATCATCGCCCGGTTTCCA
***PKM2***	F: GCTGCCATCTACCACTTGC R: CCAGACTTGGTGAGGACGATT

### Western blot assay

Cells were collected, lysed, and denatured. The Bradford method was performed to evaluate the amount of proteins. Forty micrograms of protein was separated using sodium dodecyl sulfate-polyacrylamide gel (SDS-PAGE) electrophoresis. The protein was transferred to a polyvinylidene fluoride membrane using the electrotransfer method after being blocked with skimmed milk for 2 h. Subsequently, the blots were visualized with primary antibody incubated overnight at 4°C, followed by goat anti-rabbit secondary antibody incubation for 2 h. The blots were visualized with the ECL Fluorescence Detection Kit. Photographs were taken and the ImageJ software was used to calculate the gray value of each band. The primary antibodies used were as follows: FOXP3 (ab20034, 1:1,000, Abcam), PKM2 (ab137852, 1:1,000, Abcam), and GAPDH (ab8245, 1:1,000, Abcam).

### RNA immunoprecipitation assay

The RNA immunoprecipitation (RIP) assay was conducted by adopting the Magna RIP™ RNA-Binding Protein immunoprecipitation kit (Millipore, Billerica, MA, USA). The collected cells were then lysed with lysate buffer, and the magnetic beads targeting argonaute-2 (Ago2, AB13537, Abcam) antibodies or immunoglobulin G (IgG, AB172730, Abcam) antibodies were incubated together with the RIP buffer. Then, the lysate was added and incubated at 4°C overnight. These magnetic beads were incubated with protease K, followed by total RNA isolation for subsequent qRT-PCR assay.

### Chromatin immunoprecipitation

For chromatin immunoprecipitation (ChIP) measurement, the SimpleChIP Enzymatic Chromatin IP Kit (9003; Cell Signaling Technology) was used. The cells were crosslinked in 1% formaldehyde for 10 min. Immunoprecipitation of the DNA–protein complexes was performed using the PKM2 antibody (AB137852, 1:1,000, Abcam) or rabbit IgG (Cell Signaling Technology, 2729). The bound DNA fragments were further analyzed by qRT-PCR and 2% agarose gel electrophoresis.

### Immunofluorescence

When cells grew on the flap and fused to 95%–100%, they were removed from the incubator and washed three times with 1× PBS for 10 min. The cells were then fixed with 4% formaldehyde for 20–30 min and washed three times with 1× PBS for 10 min. The cells were permeated by 0.2% Triton X-100 for 2–5 min and washed with 1× PBS three times for 10 min. BSA (5%) was used for sealing for 30 min. The sample was combined with the primary antibody and put in a wet box overnight at 4°C. After incubation of the sample with the secondary antibody in the dark for 30 min, it was washed with 1× PBS three times for 10 min. Glycerin (95%) was used to seal the film and the image was viewed under a fluorescence microscope.

### Immunohistochemical staining

Paraffin sections were dewaxed with water. The sections were incubated in 3% H_2_O_2_ for 5–10 min. The sections were washed with distilled water and soaked in PBS for 5 min. Normal goat serum (5%–10%; PBS dilution) was used for sealing. The sections were incubated for 10 min. The primary antibody was added and incubated at 37°C for 1–2 h or 4°C overnight. PBS was used for flushing three times for 5 min. An appropriate amount of biotin-labeled secondary antibody working fluid was added and incubated at 37°C for 10–30 min. An appropriate amount of horseradish enzyme or alkaline phosphatase-labeled *Streptomyces ovalbumin* working fluid was added and incubated at 37°C for 10–30 min. DAB or NBT/BCIP was used for color rendering for 3–15 min. The sections were thoroughly rinsed with tap water.

### Xenograft model in nude mice

The cells were washed with PBS and centrifuged. The cells were washed twice in a serum-free medium, and then the cells were resuspended in the serum-free medium for cell counting so that the 200-μl suspension contained 1 × 10^7^ cells.

BALB/C nude mice were purchased and raised in SPF conditions. The animals were observed every day with unlimited access to water and food. After adapting to the new environment in the animal house at 20°C–25°C for a week, each 4–6-week-old mouse was subcutaneously inoculated with HCC cells expressing stable NEAT1 and HCC cells with NEAT1 knockout in the right axillary abdominal wall. Tumor growth was observed daily, and tumor volume was measured with a vernier caliper after tumor growth. Tumor volume = (*a* × *b* ^2^)/2 (*a* represents the long diameter of the tumor, and *b* represents the short diameter of the tumor). The relative volume (RTV) of the tumor was calculated once every 2–3 days of observation. The average volume of transplanted tumors in each group was used to plot the growth curve of the transplanted tumors. Nude mice were photographed 30 days after inoculation and the tumor was removed after death. The weight of the transplanted tumors was weighed, and the tumors were surgically removed and photographed.

### Statistical analysis

The measured data were expressed as mean ± standard deviation (*x* ± *s*). The Student’s *t*-test was used for the comparison between two groups. Comparisons between three components were first made using ANOVA, and *P <*0.05 was considered significant.

## Results

### NEAT1 knockdown can inhibit the viability, proliferation, migration, and invasion of hepatocellular carcinoma cells

**Figure 1:**
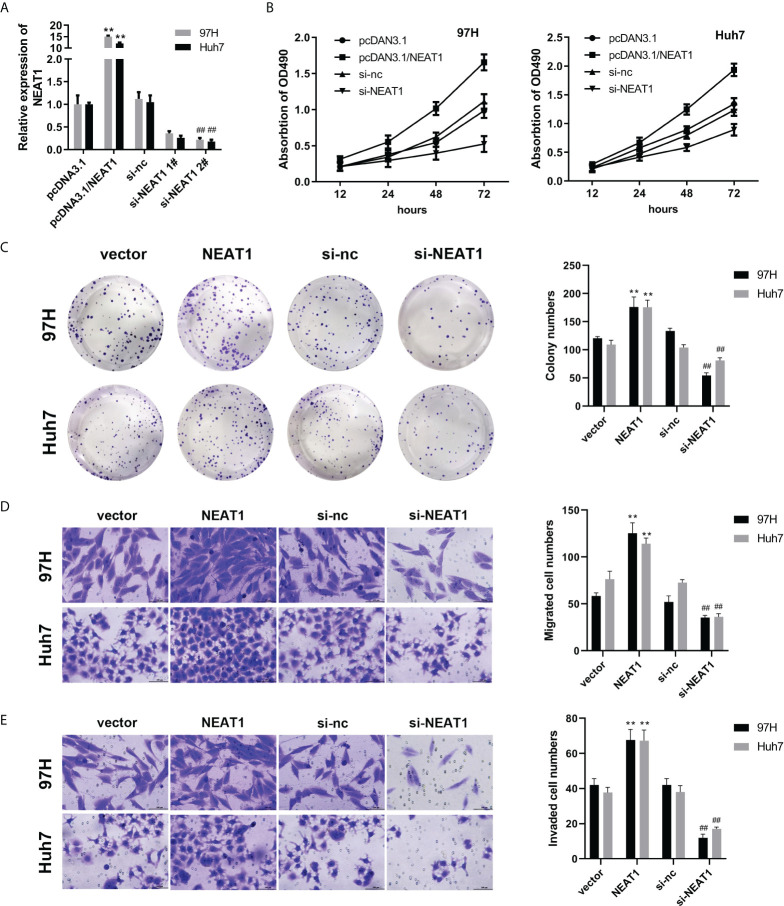
Knockdown of NEAT1 can inhibit the viability, proliferation, migration, and invasion of hepatocellular carcinoma cells. **(A)** qPCR was used to detect the overexpression or interference efficiency of NEAT1. **(B, C)** The MTT and colony formation assay were used to detect cell viability and proliferation. **(D, E)** A Transwell assay was performed to evaluate the migration and invasion of HCC cells. ** indicates *P <*0.05 compared with pcDNA3.1 or vector; ^##^ indicates *P <*0.05 compared with Si-nc.

To study the biological function of NEAT1, we first overexpressed NEAT1. Overexpression or the interference efficiency of the plasmids was detected by qPCR. Overexpressing or silencing plasmids effectively increased or reduced the expression level of NEAT1 (**Figure 1A**). Based on preliminary data, the effect of NEAT1 on the viability and proliferation ability of HCC cells was determined by MTT and the colony formation assay. Overexpression of NEAT1 accelerated the viability and proliferation of the 97H and Huh7 cell lines, while knockdown of NEAT1 inhibited the viability and proliferation of the HCC cell lines (**Figures 1B, C**). The Transwell assay was further carried out to evaluate the effect of NEAT1 on the migration ability of HCC cells. Overexpression of NEAT1 enhanced the migration formation ability of the HCC cell lines. At the same time, knockdown of NEAT1 inhibited the migration of the HCC cell lines (**Figures 1D, E**).

### NEAT1 binds FOXP3 to promote PKM2 transcription

**Figure 2:**
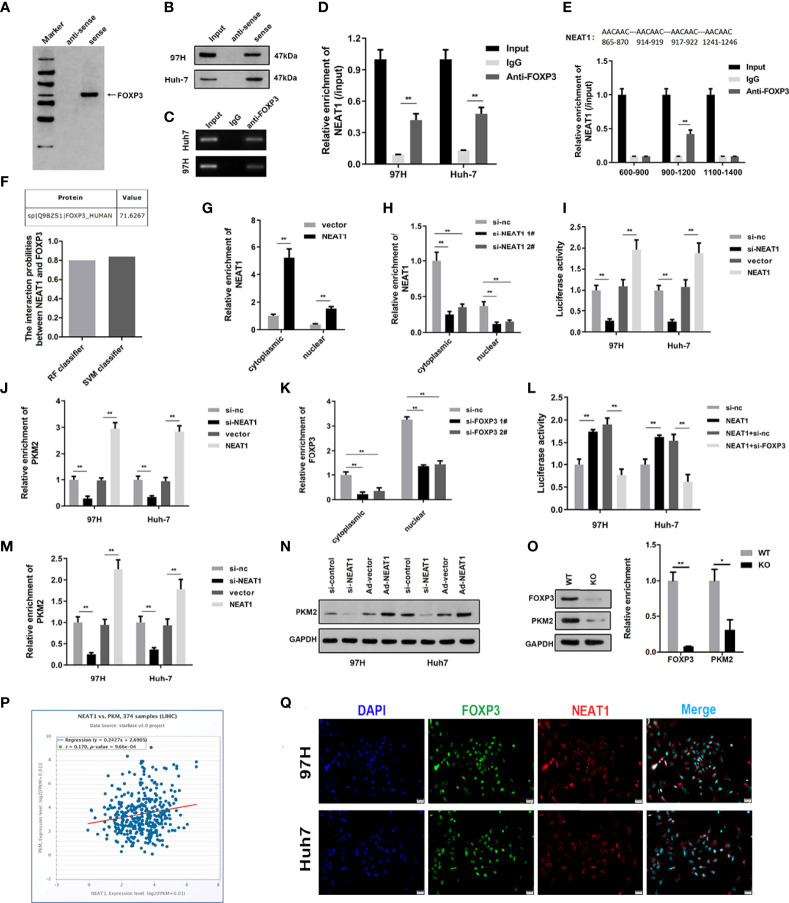
NEAT1 binds FOXP3 to promote PKM2 transcription. **(A)** RNA pulldown, silver staining, and mass spectrometry analysis were carried out to investigate the interaction between NEAT1 and FOXP3. **(B)** RNA pulldown was performed to verify the binding between NEAT1 and FOXP3. **(C, D)** RIP experiments confirmed the binding between NEAT1 and FOXP3. **(E)** Bioinformatics predicted the binding sites of NEAT1 and FOXP3, and RIP again found that NEAT1 was the binding site in the 900–1,200 fragment of FOXP3. **(F)** Bioinformatics catRAPID predicted the combination score of NEAT1 and FOXP3, proving that the possibility of the combination was high. **(G, H)** After overexpression or knockdown, qPCR was used to assess the expression of NEAT1 in the cytoplasm and nucleus. **(I)** A luciferase reporter experiment was performed to investigate whether NEAT1 overexpression or knockdown could change the transcription activity of PKM2. **(J)** A ChiP assay was performed to detect the interaction between FOXP3 and PKM2 under NEAT1 knockdown or overexpression. **(K)** qPCR was used to assess the expression of FOXP3 in the plasma and nucleus. **(L)** A luciferase reporter experiment was performed to investigate the transcription activity of PKM2 after the transfection of pcDNA3.1/NEAT1, si-FOXP3, or their combination. **(M, N)** qPCR and Western blot were used to detect the effect of NEAT1 overexpression or knockdown on PKM2 expression. **(O)** After the knockout of FOXP3 by Crispr/cas9, PKM2 expression was also weakened, confirming the regulatory relationship between FOXP3 and PKM2. **(P)** Bioinformatics analysis confirmed the positive correlation between NEAT1 and PKM2 expression. **(Q)** An immunofluorescence confocal co-localization experiment indicated the co-localization of NEAT1 and FOXP3. * indicates *P <*0.05; ** indicates *P <*0.01.

To study the downstream binding proteins of NEAT1, we used WB and mass spectrometry to analyze the binding proteins of NEAT1. The experimental results showed that NEAT1 could bind to FOXP3 (**Figure 2A**). The RNA pulldown experiment further confirmed the binding between them (**Figure 2B**). The RIP experimental results were the same as the RNA pulldown results, and the binding of NEAT1 and FOXP3 was verified (**Figures 2C, D**). Bioinformatics predicted the binding site between NEAT1 and FOXP3. The following RIP experiment showed that NEAT1 binds with FOXP3 at the 900–1,200 fragment site (**Figure 2E**). Bioinformatics analysis of the combined score using the catRAPID software showed a high probability, with the predicted value reaching 71.6267 (**Figure 2F**). After overexpression or knockdown, the level of NEAT1 in the cytoplasm and nucleus was detected to prove the efficiency of the plasmids. The experimental results showed that overexpression or knockdown plasmids could effectively promote or inhibit the expression of NEAT1 (**Figures 2G, H**). Overexpression of NEAT1 can promote the transcription activity of PKM2, while NEAT1 knockdown can inhibit it (**Figure 2I**). The ChiP experiments proved that overexpression of NEAT1 could promote the interaction between PKM2 and FOXP3, while NEAT1 knockdown inhibited it (**Figure 2J**). FOXP3 was silenced by the transfection of siRNAs. The qPCR results indicated that siRNA for FOXP3 significantly reduced the expression level of FOXP3 present in both the cytoplasm and nucleus (**Figure 2K**). Knockdown of FOXP3 reversed the effect of NEAT1 on the transcriptional activity of FOXP3 (**Figure 2L**). Overexpression of NEAT1 promoted the mRNA and protein expression of PKM2, whereas knockdown of NEAT1 suppressed its expression (**Figures 2M, N** **)**. Moreover, after the knockout of FOXP3 by Crispr/cas9, PKM2 expression was also weakened, confirming the regulatory relationship between FOXP3 and PKM2 (**Figure 2O**). There was a positive correlation between NEAT1 and PKM2 expression (**Figure 2P**). indicating the co-localization of NEAT1 and FOXP3 (**Figure 2Q**).

### PKM2 is highly expressed in HCC, and knockdown of PKM2 can inhibit the viability, proliferation, migration, and invasion of HCC cells

**Figure 3:**
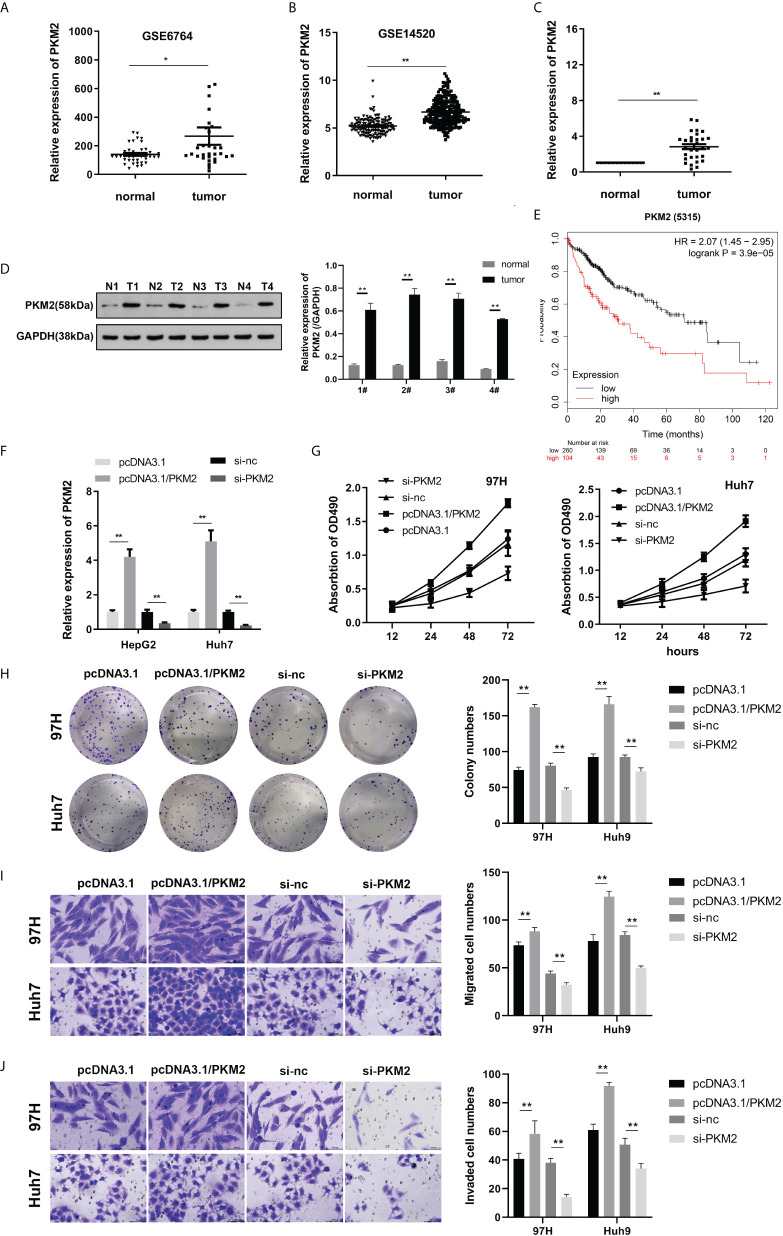
PKM2 is highly expressed in HCC, and knockdown of PKM2 can inhibit the viability, proliferation, migration, and invasion of HCC cells. **(A, B)** The expression of PKM2 based on the GSE6764 and GSE14520 datasets from the GEO database was analyzed. **(C)** The expression of PKM2 in the tissues was detected using qPCR. **(D)** Western blot was used to detect PKM2 expression in tumor tissues. **(E)** Survival analysis confirmed that PKM2 was associated with the prognosis of HCC. **(F)** qPCR was performed to detect PKM2 after overexpression or knockdown. **(G)** Cell viability was assessed using the MTT assay. **(H)** Clone formation detection of proliferation was carried out. **(I, J)** A Transwell assay was carried out to evaluate cell migration and invasion. * indicates *P <*0.05; ** indicates *P <*0.01.

Previous studies have shown that PKM2 is highly expressed during tumor formation. To investigate the effect of PKM2 on HCC, further studies were carried out. We first analyzed the expression of PKM2. The expression of PKM2 from the GEO database of the GSE6764 and GSE14520 datasets was analyzed, and PKM2 was highly expressed in HCC (**Figures 3A, B** **)**. We performed qPCR, Western blot, and immunohistochemical staining on PKM2 in the tissues of HCC patients. PKM2 was highly expressed in HCC (**Figures 3C, D** **)**. Prognostic analysis of the genes in the GSE14520 dataset showed that patients with high PKM2 expression had a shorter survival period (**Figure 3E**). qPCR was used to detect PKM2 expression after transfection of overexpressing or silencing plasmids (**Figure 3F**). Furthermore, MTT and colony formation experiments were used to detect the effect of PKM2 on HCC. Overexpression of PKM2 promoted the viability and proliferation of HCC, while PKM2 knockdown inhibited the viability and proliferation of HCC (**Figure 3G, H**). To further evaluate the function of PKM2, we used the Transwell experiment to assess the effect of PKM2 on the migration ability of HCC cells. Overexpression of PKM2 could enhance the migration formation ability of the HCC cell lines (**Figures 3I, J**).

### PKM2 knockdown reversed the function of NEAT1

**Figure 4:**
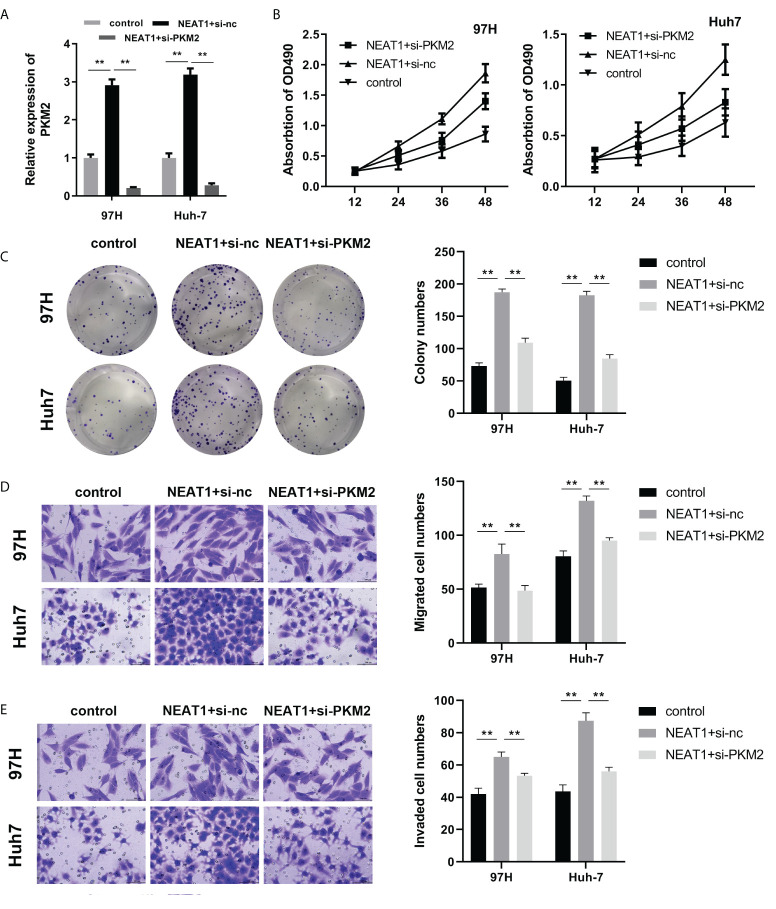
PKM2 silencing reversed the function of NEAT1. **(A)** qPCR was used to detect PKM2 expression. **(B)** The MTT assay for viability detection was performed. **(C)** A clone formation assay was used to detect proliferation. **(D, E)** A Transwell assay was carried out to evaluate cell migration and invasion. ** indicates *P <*0.01.

To find the relationship between NEAT1 and PKM2, we applied the rescue experiment to study the regulatory relationship between the two genes. qPCR was used to detect PKM2 overexpression or interference efficiency. The results showed that the overexpressing plasmid or knockdown plasmid could effectively promote or inhibit the expression of PKM2, respectively (**Figure 4A**). To determine the viability and proliferation of HCC cells, overexpression of NEAT1 promoted the viability and proliferation of HCC cells, while knockdown of PKM2 reversed the viability and proliferation ability of NEAT1 on HCC cells (**Figures 4B, C**). Overexpression of NEAT1 promoted the migratory and invasive ability of HCC cells, whereas PKM2 knockdown reversed the migratory and invasive ability of NEAT1 on HCC cells (**Figures 4D, E**).

### *In-vivo* experiments were conducted to investigate the regulatory effects of NEAT1 and PKM2 on the growth of hepatocellular carcinoma cells

**Figure 5:**
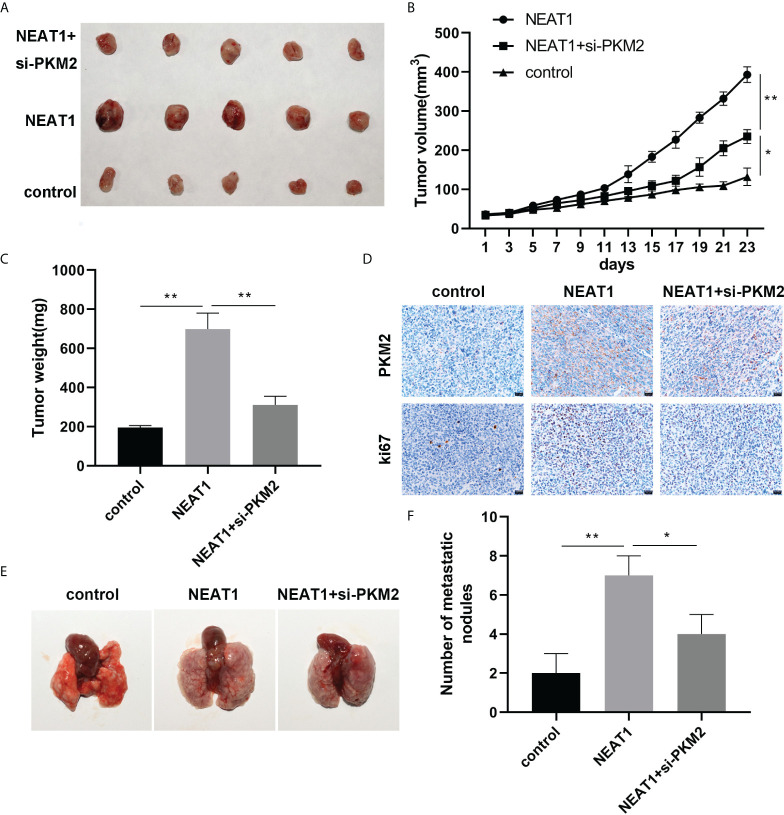
*In-vivo* experiments were conducted to investigate the effects of NEAT1 and PKM2 on the growth of HCC. **(A)** A picture of the tumor is shown. **(B)** The curve of tumor growth. **(C)** The weight of the tumors was assessed. **(D)** Histochemistry was used to detect the expression of Ki67 and PKM2 in the tumor tissues, proving the conclusion of the cell part. **(E, F)** Lung metastasis was analyzed. * indicates *P <*0.05; ** indicates *P <*0.01.

To prove the effects of NEAT1 and PKM2 on HCC, we built a nude mouse xenograft model. Overexpression of NEAT1 promoted tumor growth, while PKM2 knockdown reversed the oncogenic effect of NEAT1 (**Figures 5A, B**). The results of tumor weight were consistent with the results of tumor volume. Overexpression of NEAT1 increased the weight of tumors, while PKM2 knockdown reversed this effect of NEAT1 (**Figure 5C**). The expression of Ki67 and PKM2 in tumors was detected by IHC. NEAT1 promoted the expression of Ki67 and PKM2. Knockdown of PKM2 attenuated their expression compared to the NEAT1 group (**Figure 5D**). Furthermore, the liver metastasis of the HCC tumor was detected. As shown in **Figures 5E, F**, NEAT1 promoted the liver metastasis of HCC, while PKM2 knockdown reversed this effect of NEAT1.

## Discussion

Primary HCC is a malignant tumor all over the world (18, 19). At present, the postoperative metastasis and recurrence of HCC make the overall treatment effect still unsatisfactory. lncRNA plays a crucial role in normal human and disease development (20–22). The expression of lncRNA is tissue-specific and is usually dysregulated in many types of tumors. It has been confirmed that some lncRNAs are related to tumor recurrence and poor prognosis. lncRNA can regulate tumor suppressor or oncoprogenitor genes through epigenetic modification, shear, RNA degradation, and post-translational modification, thus participating in tumor biological processes such as tumor generation and metastasis.

lncRNA NEAT1 was initially found in chromosome II of multiple secretory adenoma type I of familial tumor syndrome. lncRNA NEAT1 was related to TNM staging, lymph node metastasis, distant metastasis, poor prognosis, and other clinicopathological features. After silencing this gene, the viability, proliferation, and invasion of tumor cells were significantly inhibited. Chai et al. reported that the high expression of lncRNA NEAT1 in ovarian cancer tissues was not only related to clinical staging and lymph node metastasis but also promoted the malignant biological behavior of tumor cells after the high expression, while the opposite result was found after NEAT1 was knocked out. In addition, Ke et al. found that lncRNA NEAT1 regulated the survival of breast cancer cells.

As in HCC, lncRNA NEAT1 has been proved to accelerate the invasion and migration of HCC cells (23–25). However, the mechanism underlying the effect of NEAT1 remains very limited. In this study, lncRNA NEAT1 promoted the viability, proliferation, and metastasis of HCC *in vitro* and *in vivo* as previously reported. Mechanically, NEAT1 can bind with FOXP3 directly in HCC cells.

FOXP3 is the most specific biomarker of Treg and is involved in the maintenance and immunosuppression of Treg cells (26). The relationship between tumor cells and FOXP3 has received increasing attention. FOXP3 is expressed in many tumor cell lines and tumor cells in tumor tissues. Related research findings found that downregulation of FOXP3 inhibited tumor cell invasion by reducing MMP-9 and MMP-2. With FOXP3 knockout, tumor cells secreted less IL-10 and TGF-β1, and T-cell survival was significantly upregulated, suggesting that FOXP3 plays an important role in malignant phenotypes, particularly during invasion and immune escape (27). In HCC, the main research on FOXP3 is still focused on the immune system. For instance, CD4^+^CD25^+^ regulatory T cells in the liver of patients with hepatocellular carcinoma were notably increased. In addition, CD4^+^FOXP3^+^ regulatory T cells activated the CD39/ENTPD1 pathway and promoted hepatic metastatic tumor growth in mice. We performed RNA pulldown and RIP experiments which confirmed that NEAT1 could bind with the FOXP3 protein directly. As a transcription factor, FOXP3 modulated the expression of numerous genes transcriptionally. We screened and found that FOXP3 may regulate the transcriptional activation of PKM2 by binding with NEAT1.

In terms of growth signals of cancer cells, there is a dynamic balance between the tetramer and dimer of PKM2 (28, 29), which is regulated by the signals of the tumor suppressor and oncogenic proteins (P53, cMYC), in phosphorylation and acetylation. Anastasiou et al. reported that increased PKM2 expression could reduce ROS production (30). The more malignant the tumor cells were, the higher the glycolysis level was, and the stronger the expression and activity of hypoxia-inducible factor (HIF)-1 were, which promoted the malignant tumor progression (31).

lncRNA NEAT1 was highly expressed in HCC cell lines. Silencing the expression of lncRNA NEAT1 can inhibit the viability, proliferation, migration, and invasion of HCC cells, and its mechanism is related to the regulation of the FOXP3/PKM2 signaling pathway. The basic research on the biological function of lncRNA NEAT1 will provide a basis for elucidating the pathogenesis of HCC and developing new therapeutic approaches.

## Data availability statement

The original contributions presented in the study are included in the article/supplementary material. Further inquiries can be directed to the corresponding author.

## Ethics Statement

All experiments were performed in adherence with the ARRIVE guidelines and the National Institutes of Health (NIH Publication, 8th Edition, 2011) guidelines for the use of laboratory animals. The animals care and experimental protocols were examined and verifed by Laboratory Animal Ethics Committee of Nanjing Drum Tower Hospital affiliated to Nanjing University School.

## Author contributions

All authors contributed to the article and approved the submitted version.

## Funding

This work was supported by the Jiangsu Province Government Foundation (No. 2016-WSW-067).

## Conflict of interest

The authors declare that the research was conducted in the absence of any commercial or financial relationships that could be construed as a potential conflict of interest.

## Publisher’s note

All claims expressed in this article are solely those of the authors and do not necessarily represent those of their affiliated organizations, or those of the publisher, the editors and the reviewers. Any product that may be evaluated in this article, or claim that may be made by its manufacturer, is not guaranteed or endorsed by the publisher.
